# Exploring barriers to diversified dietary feeding habits among adolescents in the agrarian community, North West Ethiopia

**DOI:** 10.3389/fnut.2022.955391

**Published:** 2022-12-07

**Authors:** Eskezyiaw Agedew, Zeweter Abebe, Abebe Ayelign

**Affiliations:** Center for Food Science and Nutrition, Addis Ababa University, Addis Ababa, Ethiopia

**Keywords:** adolescents, dietary habit, barriers, agrarian community, North West Ethiopia

## Abstract

**Introduction:**

Adolescence is a critical and neglected age group of the population in any form of nutritional intervention. A comprehensive study that assesses barriers that influence their diversified feeding habit is not well investigated in qualitative approaches. Therefore, this study was conducted to fill this gap by providing evidence on exploring barriers to diversified feeding habits of adolescents in the agrarian community, North West Ethiopia.

**Objective:**

The aim of the study was to explore barriers to diversified feeding habits of adolescents in the agrarian community, North West Ethiopia.

**Methods:**

A phenomenological qualitative study design was conducted among adolescents in the age group of 10–19 years, and adults in the age range of 25 to 64 years (representatives of farmers, agricultural, health, and education sectors). We conducted 24 in-depth interviews (12- adolescents, 4-health, 2- agricultural extension, 3-education, and 3-farmer representative) among purposively selected community groups. In-depth interview guides and observation checklist were utilized for data collection. The audio-recorded qualitative data were transcribed word by word into English. Finally, the translated data were exported to ATLAS ti version 7.1 software for thematic analysis based on inductive content analysis. All coded quotations, including memos written throughout the coding process, were then analyzed to identify themes inductively.

**Results:**

Dietary habit of adolescents in the study area was predominantly plant-based cereals with low protein, vitamins, and mineral contents. Adolescents have limited consumption of fruits, vegetables, and animal source foods. Adolescents with these feeding habits had suboptimal intake of micronutrients and proteins, which are crucial for their rapid growth stage. Individual-level barriers, family-level influence, dietary tradition of community, agricultural practice (poor agroforestry practice of the community, and poor agricultural practice to produce year-round diverse food items), and week multi-sectorial collaboration for nutritional intervention all influenced adolescent dietary habits.

**Conclusion:**

Multiple layers of barriers influenced the diverse dietary habits of adolescents in the study setting. These multiple and interconnected influences ranged from individual level to multi-sectorial collaboration. Nutritional interventions should be implemented at the individual, family, agricultural, and multi-sectorial levels to improve adolescents’ diverse dietary habits.

## Introduction

Nutrition has a profound impact on the current and future health of adolescents (ages 10–19 years). A sustainable healthy diet during adolescence has the potential to limit any nutritional deficits and linear growth faltering generated during the first decade of life and may limit harmful behaviors contributing to the epidemic of non-communicable diseases (NCDs) in adulthood (1–3).

Adolescence is a transitional life phase characterized by marked biopsychosocial changes capable of determining the future health status in adulthood. During this phase, dietary habits are established, and positive or negative health-related behaviors are adopted and maintained throughout adulthood (4, 5).

During the period of adolescence, body growth with a rapid increase in height, weight, psychological, and sexual maturity with cognitive development is observed which affects nutritional needs and habits (6, 7). During this period, the nutritional requirements also increase tremendously compared to the preceding years of growth. At this time the diet should provide not only sufficient calories but also essential elements and nutrients such as proteins, vitamins, and minerals (2, 8).

Adolescents’ poor dietary habits result in micronutrient deficiencies such as vitamin A deficiency, iron deficiency (anemia), and iodine deficiency. These micronutrient deficiencies have been major nutritional problems in developing countries, adversely affecting adolescents’ health and performance (9). Lack of diversified diet feeding habits increases the vulnerability of adolescents to different nutritional problems like obesity and elevated risks for type 2 diabetes, metabolic syndrome, and cardiovascular diseases (10, 11).

Adolescents consume diets that are not in line with healthy dietary patterns. For example, studies from developed countries found that adolescents consumed low intake of fruits, vegetables, dairy products, and whole grains but consumed more soft drinks and fast foods (12). This dietary habit of adolescents was shifted to developing countries and become one of the public health problems (5).

Improving adolescent dietary habits is one of the key nutritional interventions to improve their nutritional status in low- and middle-income countries (13). Adolescent dietary behaviors are influenced by a complex set of barriers that include individual, family, community level, and environmental factors (14). There was no scientific evidence on context-specific understandings of how socio-cultural and other influences affect adolescents’ dietary diversified feeding habits in the current study setting. Therefore, it is crucial to understand the barriers influencing the dietary behavior of adolescents. There was no comprehensive study on identifying barriers that contributed to adolescent diversified dietary feeding habits (15, 16). Hence, this study was conducted to fill this evidence gap among adolescents in the agrarian community, North West Ethiopia.

## Materials and methods

### Study area

North West Ethiopia consists of different districts with low land, mid-land, and high land ecology Zone. Dembecha Woreda is purposively selected for this study because it consists of all of the agroecology that can represent North West Ethiopia. Agriculture is the most important source of income, with a wide variety of food crops produced. Based on the district agricultural report data, the major cultivated crops are teff, maize, wheat, legumes (beans), and barley. This study setting has low, mid, and high land ecology zone which is suitable for fruits and vegetable production. The study area is well-known for cattle, sheep, goats, and poultry breeding.

### Study design and subjects

A phenomenological qualitative study was conducted among purposively selected adolescents within age range of 10-19 years. In addiction data was collected from representatives of farmers, health, agricultural, and educational sector focal persons by taking into consideration the principles of maximum variation. In this study adolescents in age group 10–19 years, and adults in age range of 25 to 64 years (representatives of farmers, agricultural, health, and education sectors).

### Qualitative data collection and analysis

#### In-depth interview

The in-depth interview guide was used to collect data by incorporating theoretical and practical guidelines. In-depth interviews (IDIs) were conducted to assess the multiple layers of barriers that influence the diversified dietary habits of adolescents. It was prepared to assess family-level influence, dietary tradition of community influence, cultural and religious influence, accessibility and availability of diverse diet, cost and affordability of nutritious food, seasonality influence, agroforestry practice of the community, agricultural practice, and multi-sectorial collaboration related. The IDI data were collected from different audiences through in-depth interviews with (12-adolescents, 4-health, 2- agricultural extension, 3-education sector representatives, and 3-farmer representatives) from carefully chosen community groups. The number of IDI was determined based on information saturation principles. IDI was used to collect institutional and multi-sectorial related barriers from institutional focal experts working in the health, education, and agriculture sectors. In addition to characterizing the actual type of food items in the diet of adolescent’s observation checklist were utilized for data collection during meal times.

#### Data analysis

The audio data were transcribed contextually word by word, translated to English, and saved as plain text. To realize this, the contents, meanings, and interpretation of the collected data were carefully translated based on the local context. Much of the analysis was done in the field to reduce data loss. The translated records were cross-checked by independent qualitative researcher. Finally, the data were exported to ATLAS version 7.1 software for further coding and thematic analysis. An inductive content analysis was conducted to explore barriers to adolescents’ diverse dietary habits. This enabled us to identify themes and factors influencing adolescents’ diverse dietary habits. Finally, themes were identified and coded in order to categorize, explain, and describe each participant’s perspectives. Based on the identified themes and factors, a model was developed that serves as a conceptual framework for explaining the barriers to diverse dietary habits of participants in the current study area as well as in other similar settings.

#### Trustworthiness of the study

Different measures were taken to assure the trustworthiness or validity of the research findings. Prior to the actual study, we created and tested the interview guide and observational check list. Second, data were collected from diversified study participants to assure social representativeness (adolescents, farmers, health, education, and agriculture sector representatives), and also recruited from urban and rural settings. Third, a research assistant was employed who has a master of public health and experience in conducting research and was involved in this research during transcription and coding. Fourth, member checking was done at the end of the data collection by summarizing major thematic areas raised during the interview. Research teams also reviewed and gave their comment on the report and crosscheck each other. To ensure the transferability of the findings, the study setting, study participants, and study findings were described in detail with a diverse group of population with different sex, age category, representatives from different sectors, and responsibilities. Confirmability was achieved by collecting dense (thick) data from lived experience of study participants, conducting negative case analyses, arranging for a confirmability audit, and establishing referential adequacy. The principal investigator had more than 10 years’ experience in the nutrition research area and exposure in the study setting. This preconception knowledge and skill in the study setting help the principal investigator to translate participant’s responses and views contextually while conducting this research. To minimize interpretation bias, the principal investigator focused on the idea of participants, recruited different participants from different social segments, documents reviewed by peers and research advisors, and focused on the context idea of participants. All the research team checked the consistency of the interpretation with the quotations taken from study participants.

## Compliance with ethical standards of study

Ethical approval was obtained from the College of Natural and Computational Sciences institutional review board (IRB) with IRB number CNCSDO/188/14/2021, Addis Ababa University. Besides, a permission letter was secured from the Dembecha Woreda health office. The aim, potential risk, benefits, and confidentiality were made known to respondents in the informed consent form. Additionally, participation was voluntary based and we gave them the full right to refuse or accept to participate. The anonymity and confidentiality of the respondents’ data were assured. Informed oral consent for participants under 18-year-old adolescents was taken from their parents or legal guardians. Direct informed consent was taken from adolescents who were above 18 years and adults study participants.

## Result

### Diversified dietary feeding habits of adolescents

The existing dietary habit of adolescents was predominantly cereal-based with low protein, vitamins, and mineral content. From the direct observation of selected adolescent’s meals, the diet composition consists of *injera* (a traditional cereal-based dish made mainly from the mixture of maize, barley, wheat, and *teff* flours through household traditional fermentation and baking methods).

*“The adolescent diet was almost in observed house hold consists of traditional diet injera with wote sauce in all meal times”* (*Female, diet observer data collectors, 27 years).* The same observation result was confirmed by the second data collector *“in my observation during all meal time of adolescents, there was no variation of diet*” *(male, diet observer, data collector, 31 years).*

Concerning the sauce (*wote or shero*), it was made from flour roasted legumes mainly beans in rural area and peas in urban areas, red paper, spices, and oils. This traditional diet *Injera* with shero was the major diet of adolescents including all family members. All family members eat *injera* with shero almost in all meals. This shows that adolescents did not consume a diversified diet.

*A 16 years of old male respondent said that “my daily meals during breakfast, lunch, and dinner was injera with wote sauce made from mixed salt and red paper” (IDI, male, 16 years).*

In terms of daily meal schedules, adolescents eat only lunch and dinner, but breakfast consumption is low due to fasting influence and lack of ready-to-eat food in the morning. Adolescents did not have the habit of snake consumption. Adolescents’ feeding habits result in insufficient daily nutrient intake or recommended dietary allowance.

*A 17 years of old female participant said that “I had a breakfast eating habit during non-fasting, but I do not eat at time of fasting” (IDI, female, 17 years). Similar response was reported from other male adolescents, I prefer food items like potatoes, legumes, fruit, and vegetables but I do not get access to this variety of food items in my family. Because in my area there is no accessibility of a variety of food items (IDI, male, 16 years).*

Food from animal sources was consumed during major religious festivals in the study area. This religious festival was celebrated in the local area collectively by all segments of the population including adolescents. During this celebration, meat and traditional alcohol drinking mainly tela (a fermented alcoholic beverage) were consumed. This is a time when adolescents get access to eat animal sources of food.

*I rarely eat eggs, meat, and milk during none fasting times and most of the time I eat during fasting season beginning and at the end during religious festivals such as chris-mass, epiphany, Easter, and New Year (IDI, female, 16 years).*

Fruit and vegetable consumption habit of adolescents was very low, only few green vegetables mainly cabbage was consumed at time of rainy sessions of the year. This is due to the lack of access and seasonal influences fruit and vegetable consumption among adolescents.

This supported with response of a 16-year-old male adolescent “*I know a healthy diet but due to limited food items in my home, I do not consume diversified food items. I have a preference to eat fruit and vegetables” (IDI*, male, 16 years).

### Barriers that influence diversified dietary feeding habits of adolescents

After qualitative data analysis, five main themes were identified after categorizing the codes into a category of similar contents of themes. The major identified barriers to diversified dietary feeding habits of adolescents consists of individuals, family-level influence, community-level influence, agriculture practice (poor and traditional agriculture, agroforestry, irrigation, and agroecologic influence to produce year-round diverse food items), and weak multi-sectorial collaboration for nutrition intervention (Table 1).

**TABLE 1:** Barriers domain, category, and specific barriers that influence diversified dietary habits of the adolescents, North West Ethiopia.

Domain category	Barrier category	Reported barriers	Implication on a diversified dietary habit of adolescents
Individuals level influence	Low consumption of animal sources foods, fruit and vegetables	✓ Poor appetite on animal source food consumption ✓ Skipping breakfast, and snack daily meals ✓ Lack of access to fruit and vegetables	Inadequate consumption of diversified diet for daily recommended dietary allowance (RDA)
Family level influence	Lack of accessibility, and availability of diverse diets at the household level Lack of family support for adolescent diet Lack of alternative income High cost of animal sources diet, and fruit	✓ Selling of quality animal sources food like eggs and poultry, sheep and milk products like butter instead of consumption. ✓ Low family income ✓ Family do not give priority to adolescent’s diet ✓ Low attention for a diversified diet ✓ Food provision preference for male adolescents	Adolescents are dependent on their families for their daily meals and they are not autonomies to consume diversified food items. Family food environment directly influenced adolescent dietary habit to low dietary consumption of animal source foods, fruit, and vegetables.
Community-level influence	Cultural and religious influence of community on diversified diet	✓ Community do not give value for diversified feeding. ✓ Religious influence ✓ Cereal-based diet feeding habit of community as common practice ✓ No preference for adolescent diet	This cultural tradition, value on diet, and fasting influence limit adolescents to eat animal sources food. It also influences and shapes current and future adolescent dietary habits and norms to eat only cereal-based diet all the times.
Agricultural practice	Low production of diversified food items due to dominant cereals based food production habit in the area.	✓ Lack of irrigation land and water access. ✓ Lack of agro ecologically adapted agroforestry plants in the area. ✓ High desire of farmers to plant eucalyptus trees instead of agroforestry plants which produce fruit and vegetables. ✓ Lack knowledge among farmers on agroforestry plants cultivation. ✓ Poor home gardening practice, and climatic (seasonality) influence to cultivate diversified food items	Lack of access and availability of diversified food consumption at households and markets directly influence adolescents’ diversified food items year-round.
Multi-sectorial collaboration and lack self-standing structure to implement nutrition intervention	Poor collaboration, and coordination among health, education and agricultural and other sectors Absence of nutrition human power or officer assigned in health, education, and agricultural sector. Lack of self-standing structure to implement nutrition interventions programs for adolescents and other target groups in health, education, and agricultural sector.	✓ Lack of trained nutrition profession at all level ✓ Work load for assigned profession at Woreda, Zonal and Region level ✓ There is no monitoring and evaluation mechanisms for implementation of multi-sectorial action for nutrition. ✓ Lack of budget/finance for nutrition ✓ Lack of nutrition education at school and village label. ✓ Lack of school-based nutrition intervention. ✓ Lack of non-governmental organization (NGO) support on adolescent nutrition and diversified food items production.	Poor adolescent knowledge on diver’s dietary practice forced adolescent’s to eat monotonous food item. Lack of accessibility and availability of diversified food items. Brings limited consumption of diversified diet.

### Emerging conceptual framework of barriers to diversified dietary feeding habits of adolescents

**FIGURE 1:**
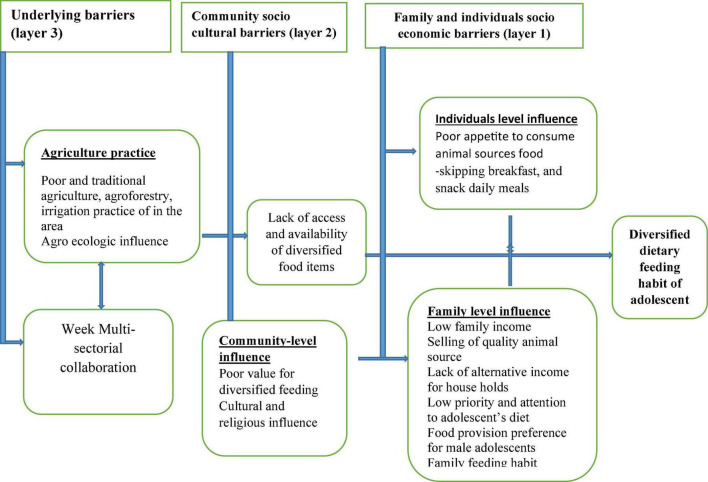
Conceptual framework of barriers for diversified dietary feeding habit of adolescents, 2021.

An inductive content analysis was conducted to explore adolescents’ lived experiences regarding their diversified dietary habits and barriers. After categorizing similar contents, five themes were identified with three major layers of barriers to diversified dietary feeding habit of adolescents (Figure 1).

#### Individual-level influence on diversified dietary habits of the adolescents

Adolescents were more aware of the importance of eating a diversified healthy diet and its long-term consequences for a latter age. Adolescents make good dietary choices if they have sufficient nutritional knowledge, including nutritional recommendations and information on benefits and risks related to foods or dietary behaviors.

A 16-year-male participant said “*I prefer food items like potato, legumes, fruit, and vegetables, but I do not get access to this variety of food items in my family home. Because in my locality we do not have access a variety of food items” (IDI, male, 16 years).*

Adolescents perceived that the consumption of animal sources food had health benefits but they ignored it in their daily meals. This was due to poor appetite for animal sources food items, especially girls who had a poor appetite to eat animal’s sources food such as meat, eggs, dairy, and dairy products.

This statement is supported by IDI of 17-year-old female adolescents “*I do not like to eat animal source foods like eggs, milk and meat since my child hood. I am not comfortable psychologically to eat animal source foods” (IDI, female, 17 years).*

In addition, skipping meals habit and food restriction practices were the major berries for adolescents’ dietary habits. A 14-year-female adolescents report that “*I do not have eating habit of breakfast due to shortage of time to prepare my breakfast; instead first I wash my hand and face, immediately I go to school” (IDI, female, 14 years).*

Adolescents stated that they consume a cereals-based diet despite awareness of diversified dietary habits. This indicated that there was a clear gap between knowledge and actual practice of diversified dietary feeding habits of adolescents.

This is supported by IDI of 16-year-old male adolescents’ stats that “*I know a healthy diet but due to limited food items, I do not consume diversified food items. I have a preference to eat fruit and vegetables”*(IDI, male, 16 years). The same idea was supported by other adolescents by narrating as *“I prefer to eat vegetables, and animal source food items like milk, egg, and meat. The barriers that prevent me from eating a diversified diet were family problems, lack of access to different food items in my home” (IDI, male, 16 years*).

#### Family-level influence on diversified dietary habits among adolescents

Family feeding behavior was considered to have both positive and negative influences on adolescents eating habits. Home availability and accessibility of food items like; fruits, vegetables, and animal food sources were the major barriers to adolescents’ diversified dietary habits. Family dietary habits were identified as an important factor influencing adolescents’ feeding habits. Many adolescents reported that their food choices were limited based on family meal frequency and type of food available what they eat. Eggs were provided when family members get ill and for young children.

This finding was narrated by IDI of 17-year-old male adolescents “*My feeding habit is similar to my family, in my family, there was no priority for adolescent diet mainly quality diet was given for young children and sick individuals. We eat a large portion diet but the quality of diet is low which is predominantly cereal-based injera and wote” (IDI, male, 14 years).*

At the family level, there were no any diet considerations for adolescents at household levels. Most of the time nutritious diet was provided for young children and sick individuals. At the family level, there was no special support for adolescents’ diet.

This was supported by 14-year-old male adolescents “*My family does not give attention to a balanced diet*; *we eat what we found” (IDI, male, 14 years)*. The same idea was supported by other adolescents and narrated as “*even I had animal source food items in my family level, our feeding habit is poor due to lack of awareness, and my family do not give attention to feed diversified food items for adolescents” (IDI, female, 16 years).*

Adolescents’ diversified dietary habit was influenced by the accessibility and availability of diversified food items at home. The adolescents’ diversified feeding habits were influenced by their home food environment because they eat what was served and available at home.

This is supported by 16-year-male adolescents as “*I have knowledge on healthy diet, but due to limited food items, I do not consumed diversified food. I know a healthy diet but due to limited food items, I do not consume diversified food items. Even I have a preference to eat fruit, and vegetables” (IDI, male, 16 years).*

There was relatively good accessibility, and availability to poultry and eggs, but few of them had access to meat, dairy, and dairy products. But adolescents and other family members do not consume frequently these quality food items. Rather, they sold eggs and poultry to the local market instead of eating.

This statement was supported by 17 years of female adolescents stating that *“I have eggs access at home, but my family sold it to a local market”* (IDI, female, 17 years). Similar idea was reflected by 45 farmers “…*I have animal like sheep and poultry, but I do not have a habit of eating eggs, and meat in our home. Because, I sold it to market to get money to pay for land rent to cultivated cereals food items and to buy agricultural input like fertilizer”(IDI, male farmer, 45 years).*

#### Dietary tradition of community influence on a diversified dietary habit of adolescents

The usual diet of the community was dominantly cereal-based with limited and infrequent consumption of animal source foods. There is consumption habit of animal source foods at the time of cultural and religious festivals. Regarding green vegetable consumption, there is seasonal consumption of cabbage in the summer season.

This was supported by 14-year-male adolescents by narrating as “*We eat large portion but low-quality diet which is predominantly cereal based traditional diet. Our community do not give attention on balanced diets, we do not wary about its quality” (IDI, male, 14 years).*

The existing dietary practice of the community was almost monotonous. The community does not give value to the quality of food that should be consumed at the household level.

A 16-year-old female adolescent narrated that “*The rural community does not consider to eat diversified food items” (IDI, female, 16 years).* Similar findings were reported by another adolescent, who stated, “*Even we had animal source food items in our home, but our feeding habit is poor due to lack of awareness” (IDI, male, 14 years).*

The community had a long tradition on the consumption of animal source food feeding habit only at the time of religious festivals and during local cultural ceremonies like weddings.

A 14-year-male adolescent reported that “….*I only eats animal source foods during religious festivals and in some cultural ceremonies” (IDI, male, 14 years*).

#### Agricultural and agroforestry practices of the community

The community livelihood was agrarian with predominant production of cereals (teff, maize, barley, and wheat), legumes (beans and pea), and tubers (mainly potato) in North West Ethiopia. Three agroecologic climatic zones existed in the study setting which is suitable to produce different fruits and vegetables. But the agricultural practice is rainfall dependent. Dominantly cereal crops were produced once a year. Irrigation is not practiced in the area to produce diversified food items year-round.

This finding was supported by 53-year-old farmer *“some farmers practice agroforestry plants cultivation for fruit production like mango, and avocado but apple tree plantation was not practiced by the local farmer”(IDI, male farmer, 53 years).*

There were limited cultivation agroforestry plants for fruit and vegetable production in the local area. This poor practice is due to the lack of knowledge of farmers on the importance of agroforestry plantation, low agricultural support, and lack of access to agroforestry plant trees for farmers.

A 38-year-old farmer said that “*Our agricultural system is dominantly traditional. Even the agricultural extension workers do not fully support on how to cultivate locally adaptable fruit and vegetables” (IDI, male farmer, 38 years*).

Poor agricultural sector support for the development of irrigation water resources and lack of access to agroforestry plant materials were the major barriers identified in the study area that limit farmers from the production of diversified fruit and vegetables for consumption.

A 29-year-old male agriculture extension worker said that “…*The agro ecology Zone are dry and no access to irrigation. In addition there is no access to agroforestry plant in the area. There was no agricultural office and other none governmental organization (NGO) that provide support for agroforestry fruit and vegetables production” (IDI, male, agriculture extension, 29 years*).

The agricultural land was covered by eucalyptus trees in all agroecology areas in the study setting. This tree is not agroforestry and does not consume as fruit or vegetables. On the contrary, it reduces land size and soil fertility, reducing cereal, fruit, and vegetable production in the areas. This trend of eucalyptus tree plantation expansion was due to the lack of alternative agroforestry plants that were adapted for each agroecology zones.

This finding is supported by 53-year-old farmer “*I have interest to cultivated agroforestry plants but, I do not get access agro ecologically adaptable agroforestry plants species” (IDI, male farmer, 53 years).* Further, there was no regulatory enforcement on farmers to prevent this massive plantation of farm land by eucalyptus trees. The same finding was reported by 29 years agricultural extension worker “… *due to the fact that eucalyptus tree is easily accessible at local area, the tree do not need much water for growth” Now, this is a time of rainy season but almost all farmers plant Eucalyptus tree (IDI, male agricultural extension worker, 29 years).*

#### Weak multi-sectorial collaboration and poor implementation nutrition strategy

There is a gap in improving diversified dietary habits of adolescents in the study setting. There was no planned nutrition education delivered regularly for adolescents at household and school levels. The absence of organizations that support adolescent diets, school-based nutrition intervention to promote adolescent diets, and lack of integrated agricultural and health sector support was the identified multi-sectorial collaboration barriers for the production of diversified food items. These poor multi-sectorial collaborations bring poor access and availability of diversified food items in the study area.

This finding is supported by 35 years school directors “…… *These days, there was no any school based nutrition intervention on adolescents” (IDI, school director, 35 years).* Similar idea was reflected by a 48-year-old farmer *“Even the agricultural extension workers do not fully support on how to cultivate locally adaptable fruits and vegetables” (IDI, male farmer, 48 years).*

Nowadays in Ethiopia there was a proven strategy for the implementation of nutrition-sensitive and specific interventions. Mostly interventions were implemented for under five and under two children. There was nutrition promotion at the school level for the production of garden for the production of vegetables and fruits. At this time the program did not continue due to a lack of input, nutrition education, and coordination.

This finding was supported by the elementary school director “*lack proper orientation and education to students, lack of integration between school and health center or office” (IDI, male, 32 years, school director).* The same idea was reflected with other school directors *“we did not implement school based garden for production of vegetables as model in our school, due lack of water access” (IDI, male, 29 years, school director).*

The other barriers that influence adolescents’ diversified diet feeding habits was the absence of nutrition professional recruitment at all levels of health facilities (Regional, Zonal, Woreda, Health centers, and Hospitals). At all levels of health care management in the Ethiopia nutrition programs were implemented mainly with the integration of maternal and child health services (like immunization, and child health). This approach to healthcare delivery brings overload for healthcare workers to deliver nutrition intervention for adolescents and target population.

*”To deliver nutrition intervention for adolescents and other target population we face limited human resources at regional, Zonal and Woreda level. Only one person was assigned to coordinate nutrition intervention at the community and health facility level along with other child health services”* (IDI, regional nutrition focal, 52 years).

## Discussion

Almost all interviewed adolescents frequently report limited consumption habits of animal sources food like eggs, meat, dairy and dairy products as well as fruit and vegetables. Breakfast is the most important daily meal, but neglected more by children and adolescents (17).

In addition, adolescents had meal skipping habits of breakfast and snacks due to fasting time influence and lack of accessibility of prepared food items to eat in the morning at house hold level. This feeding habit of adolescents exposed them to macro and micronutrient deficiency. Similar feeding habits of adolescents were practiced among adolescents who live in developing countries (1, 4, 18).

In this study, diversified dietary habits of adolescents were influenced by complex and interrelated barriers. This study identified five major themes of barriers which consist of individual, family level of influence, community-level influence, poor agricultural practice (lack of agroforestry and irrigation, and agroecologic influence to produce year-round diverse food), and week multi-sectorial collaboration for nutrition intervention. Similar results were identified in other studies as dietary feeding habits of adolescents were influenced by multiple and interconnected factors which consist of socio-economic and environmental barriers (13, 19).

Among these factors, individual-level influences like gender and socio-economic status of adolescent families were the major barriers that influence diversified dietary habits. This is due to the influence of gender as one of the social norms for intra-household food distribution (14, 20, 21). Similar evidence indicated that food choice in general is a complex process that depends on the culture and can be influenced by different factors such as personal, social, economic, and emotional (22). Sociocultural and socio-demographic factors were playing a crucial role in shaping the dietary patterns of adolescents (23–25). In addition, socio-cultural and economic factors play an important role in determining the food preferences and dietary habits of adolescent girls (26).

Adolescents perceived that the consumption of animal sources food had a health benefit, but they ignored their daily meals. This was due to poor appetite for animal sources food items, especially girls were more affected in this respect. This physiological influence toward animal sources food eating habits brings negative effects on adolescents’ dietary intake from animal source food items which expose them to micronutrients’ deficiency and undernutrition (6, 27).

Family dietary habits were identified as important in influencing adolescents’ feeding habits. Many adolescents reported that their food choices were limited based on family meal frequency and type of food items. Eating is a social act, and social networks and family can affect their food choices (22). Other family-level influences such as lack of priority given for adolescent diet and a lack of attention for a diverse diet influenced adolescents’ dietary habits. At the household level, women are expected to give preference to their husbands in the distribution of the quantity and/or quality of food. This leads to low caloric and insufficient micronutrient intake for adolescent girls and adult women (14, 19, 28). These family-level barriers influence adolescent feeding habits negatively as they are dependent on their family. This is due to the effect of family control, counseling, and involvement in dietary patterns in influencing adolescent eating behavior (13, 29).

Adolescents’ dietary habit was influenced by the accessibility and availability of diversified food items at home. Adolescents eat what is served and available at home. As a result, the feeding habit of the whole family was influenced by the food accessibility of a variety of food items. This is due to the effect of access to the food environment playing a critical role in the diets of low-income populations (30–32).

At the community level, diversified dietary feeding influences such as traditional consumption habits of monotones cereal-based dietary practice, wrong community value toward diversified diet, fasting influence on diversified diet and meal frequency, selling of nutritious diet to market due to lack of alternative income, and high cost of quality diet (meat, eggs, and fruit) had a direct impact on adolescents’ diversified dietary habits in the current study setting. This is because adolescent food choices are influenced by price due to limited household budget (33, 34). Certain healthy foods were preferred by adolescents but, there were no frequent consumption habits due to high prices of these foods (26).

The community consumed animal source foods mostly at the time of religious festivals and in some cultural ceremonies such as a wedding. Religious dietary rules affect food and nutrition security, and health by affecting the quantity of food consumed, dietary diversity, and the intake of nutrient-rich foods (35, 36). In addition to community cultural influences, environmental factors influenced adolescent dietary feeding practices. This is due that the fact that cultures will evolve parallel patterns of food behavior and diet under similar environmental settings of the community (14, 37).

Local agricultural practices of the community were the major berries that influence adolescents’ diversified dietary habits, due to the low cultivation of diversified food items. The community livelihood was agrarian with predominantly production of cereals crops with seasonal cultivation of some vegetables like cabbage, chilies, and onions. This traditional cereal crop-based agricultural practice of the community brings to the low accessibility of fruit and vegetables for adolescents. Due to this, the feeding habit of the whole family was influenced by home food availability (19, 30).

There was no existing practice of agroforestry plant cultivation for fruit and vegetable production in the study area. This is due to poor knowledge of farmers on the importance of agroforestry plantation, lack of agricultural support, and lack of access to agroforestry plant trees for farmers.

Fruit tree-based agroforestry system has a great role for the smallholder farmers in improving their livelihood and generating income (38).

There was no nutrition education regularly delivered for adolescents at household and school levels. In addition, there is no governmental organization support for promoting adolescent diet, lack of school-based nutrition intervention, and lack of integrated agriculture and health sector support for the community to cultivate diversified food items to enhance the diversified dietary habit of adolescents and family members as well. This lack of multi-sectorial collaboration results in limited access to and availability of diverse food items in the area. As a result, the dietary practice among adolescents is compromised (39, 40).

## Strengths and limitations of the study

This study try to explore multi-level barriers on diversified food consumption among under studied and overlook adolescents population group in area of nutrition intervention and investigation. The study address barriers from different layers of influencing factors starting from micro level factors up to agricultural and multi-sectorial factors. As a limitation, because it is a qualitative study, it does not quantify the prevalence of diverse food item consumption.

## Conclusion and recommendation

The diversified dietary habit of adolescents in the study area was predominantly plant-based cereals with low protein, vitamins, and mineral content. Almost all adolescents have a limited intake of fruits, vegetables, and animal source foods. As a result, adolescents had a suboptimal intake of micronutrients and proteins that are crucial for this fast stage of growth. In the study setting, multiple layers of influence or barriers on a diverse dietary habit of adolescents were identified. These multiple and interconnected barriers range from individuals level barriers to multi-sectorial collaboration for nutrition intervention.

Adolescent nutrition intervention that focuses on multiple layers of influence should be implemented by taking the identified contextual factors into account in order to improve adolescents’ diverse feeding habits. Improving household agricultural practice to grow and cultivate a variety of food items by implementing home gardening and agroforestry practice. Delivering nutrition education, designing agroforestry plantation for promoting adolescents and their family’s accessibility and availability of diverse food items for sustainable production and consumption diversified diet.

## Data availability statement

The original contributions presented in this study are included in the article/Supplementary material, further inquiries can be directed to the corresponding author.

## Ethics statement

The studies involving human participants were reviewed and approved by Ethical approval was obtained from College of Natural and Computational Sciences Institutional Review Board (IRB) with IRB number CNCSDO/188/14/2021, Addis Ababa University. Written informed consent to participate in this study was provided by the participants or their legal guardian/next of kin.

## Author contributions

EA conceived and designed the study, analyzed the data, and drafted and prepared the manuscript. ZA and AA contributed to the designing methodology and editing the manuscript. All authors contributed to the article and approved the submitted version.
