# Supplementary material for: Exploring barriers to diversified dietary feeding habits among adolescents in the agrarian community, North West Ethiopia
Source: Front Nutr. 2022 Dec 7;9:955391. doi: 10.3389/fnut.2022.955391 (PMC9768322; doi:10.3389/fnut.2022.955391)
Supplement: Supplementary file 1 [file Data_Sheet_1.pdf]

Data Sheet 1 -transcription from in-depth interview of respondents

In-depth interview 1 (Rural resident, Male adolescents, age 16)

Q1. What is your food preference and eating habit?

Response: - I prefer food items like potato, legumes, fruit and vegetables but I do not get access to this variety of food items in my family. Because in my area there is no accessibility of variety of food items. These food items are not grown due to climate variation for growth and cultivation. Mainly I feed food items like *shero wote* (made from legumes most of the time beans) and *injera* (made from mixed cereals) and potato. In my area there was sessional production of vegetables like chills, cabbages and some fruits. I eat almost similar diet during breakfast, lunch and dinner times but sometimes I eat brads. In my home the only accessible food during summer session is cabbage and chills but cereals and legumes are available year round.

Q2. What type of animal sources food do you have access and your consumption habit?

I had egg access in my family but mainly we sold it to the local market. In my family I eat eggs occasionally during at the beginning and end of fasting times. We have dairy and dairy products in my family; but I do not like to drink and consume any dairy products from child hood up to now's. I eat meet at time of ceremony. I eat similar food items in all meals. If I get access I prefer fruit and vegetables.

Q3. What is healthy diet for you?

I know about healthy diet but due to limited food items I do not consumed diversified food items. I have preference to eat fruit and vegetables. My eating habit is based on my family food preferences. The barriers that preventing from eating healthy diet were family problems, lack of variety and accessible food items in my home.

The importance of health diet was it helps to regulate body growths and function. Some of the healthy diet are milk, fruit, vegetables and unhealthy diet as potato.

Do you have feeding habit outside home?

I did not eat out of my home.

Q4. Who get primarily food from your family member?

In my family the prepared food was given for head of house-hold (father) and no food preferences among other family members.

Q5. Does your family and peer influence your dietary choice?

No my peer is not influence my food preferences and feeding habit. My feeding habit is similar to my family.

Q6. Why do not you consume various food items?

In my family there is lack of diversified food items and fasting periods.

In addition, there is cultural influence in community due to long tradition on consumption habit of monotones food items.

Q7. Do you have food consumption habit outside your home?

I had no consumption habit of food outside home.

In-depth interview-2 (Rural resident, Female adolescents, age 17)

Q1. What is your food preference and eating habit?

Response- my food preferences for me was injera with wote, and brad. I dis-like animal source food like eggs, milk and meet starting from childhood up to now. I feel bad and psychologically I do not accept these food items. I had no breakfast feeding habit. I eat similar food during lunch and dinner time except sometimes I eat potato as an additional food items.

Q2. What type of animal sources food do you have access and your consumption habit?

Response- I did not get eggs, milk and fruits; due to lack of commitment to cultivate these food items. Even our hen laid eggs, my family sold to market instead of consumption. I only eat eggs at beginning and end of fasting time of the year. I eat at time of breakfast mainly brad, at lunch and dinner Injera with wote (sauce made from legumes powder). I had no sank consumption habit.

Q3. What is Healthy and Unhealthy diet?

Response - healthy diet are fruit, protein content food and milk whereas unhealthy diets like local alcohol like *arki, tella* and meet. Importance of healthy diet “To be healthy”.

Q4. Who get primarily food from your family member?

Response - First to my father and second for my brothers eat before other family member. Females eat at the end the remaining cooked foods.

Q5. Does your family and peer influence your dietary choice?

Response - I do not face peer influence on my feeding habit

Q6. Why do not you consume various food items?

Response - due to lack of access of variety food items. Even I can access eggs, but my family sold it to local market.

Q7. Do you get nutrition education at schools or village level?

No, I do not get sufficient education at school.

Q8. Do you have food consumption habit outside your home?

No, I do not eat ant food out of my home.

In-depth interview -3 (Rural resident, Male adolescent, age 14)

Q1. What is your food preference and eating habit?

Response- I prefer food that build my body, provide energy like honey and legumes, lentil and beans. We cultivate cabbage at winter session for selling and household consumption purpose.

Q2. What type of animal sources food do you have access and your consumption habit?

Response -There is limited access of animal sources food in my family. I only eat animal sources food items during the beginning and end of fasting session.

Q3. What is healthy diet for you?

Health diet are cabbage, legumes, and barley; whereas unhealthy diet such as sweet foods, alcohol (local *arki)*.

Q4. Who get primarily food from your family member?

In my family there were no such variation on food preferences among family member but my father get attention relatively.

Q5. Does your family and peer influence your dietary choice?

I had no influence of peer and I do not hear my friends if he motivates me to drink local alcohol.

Q6. Why do not you eat diversified food items?

Even I had animal source food items in my family level, but our feeding habit is poor due lack of awareness, we rural community do not give attention on feeding of diversified food items for adolescent. We eat large portion diet but the quality of diet is low which is predominantly cereal based injera and wote. Habitually my family including we eat diet like bred, injera (mixed cereals).

My family do not give attention on balanced foods, if the food is full our stomach we do not wary about its quality. my family sold eggs at market. Eggs is provided when family member get illness and for young child. I drink milk if there is no fasting. In rural area our community do not give attention on balanced diet, we eat what we found.

Q7. Do you get nutrition education at schools or village level?

No I do get not sufficient education at school.

Q8. Do you have food consumption habit outside your home?

No I do not eat out of my home.

In-depth interview 4- (Rural resident, female adolescents, age 15)

Q1. What is your food preference and eating habit?

Response- I prefer to feed variety of fruits and food items every day. But, sometimes I get only limited food items like chills, carrot, cabbages and others by purchasing from the market. At list I eat vegetables once per week. My family do not produce any food items at farm land we eat by buying from the market. Manley my mothers and older sister engaged in food preparation. I do not involve in food preparation

instead I look after animals in the field. My family purchase fruit and vegetables 1or 2 times per week from the market.

My habitual daily meals during at breakfast I eat bred. I eat injera with wote sauce, at lunch time and dinner.

Q2. What type of animal sources food do you have access and your consumption habit?

I occasionally eat eggs and drink milks. Mainly the eggs are sold to market to cover different fees.

Q3. What is Healthy and Unhealthy diet?

Eating vegetables and animal source food items like milk, egg and meet. Healthy diet is important for healthy life. Whereas unhealthy diet- like drinking alcohol and chewing chat.

Q4. Who get primary food from your family member?

In my family my father and my brother feed first from all family member due to they move early for agricultural work.

Q5. Does your family and peer influence your dietary choice?

My peer did not influence me on my food preference; but when I hear variety of food eating practice from my peers I told to my mother to prepare for me.

Q6. Do you get nutrition education at schools or village level?

No I do not get any nutrition education.

Q7. Why do not you consume various food items?

Due to lack of cultivating vegetables and sufficient income to purchase from market. In Addition-I dislike to eat meet because my family did not like eating meet.

Q8. Do you have food consumption habit outside your home?

I do not eat out of my home.

In-depth interview -5 (Rural resident, Female Adolescents, age 14)

Q1. What is your food preference and eating habit?

Response- I prefer to eat food that prevent disease, give energy and build my body. But I do not get access these food items due to lack of accessibility of these food items in my family. Some food items like cabbage is found in summer season. Mostly my family cultivated cereals crops.

Q2. What type of animal sources food do you have access and your consumption habit?

Sometimes, I eat eggs at time of fasting period ending and beginning. But, most of the time we sold it.

Q4. Who get primary food from your family member?

In my home my fathers and brothers feed first from all family member due to they move early in the morning for farming.

Q5. Does your family and peer influence your dietary choice?

My eating habit is not influenced by peers. my feeding habit is similar to my family.

Q7. Why do not you consume various food items?

I do not eat diversified food items due to lack of accessibility of variety of foods in my home. Even eggs are available in my house, we sold it to market instead of feeding. I and my family eat eggs during cultural ceremony, and at the begging and end of fasting.

My daily meal habit similar at time of breakfast, lunch and dinner.

Q8. Do you have food consumption habit outside your home?

No, I do not eat yet.

In-depth interview -6 (Rural Resident, Male Adolescents, Age 16)

Q1. What is your food preference?

Response- I prefer fruit, energy dense food and some the food are found in my family occasionally. Sometimes I got vitamin sources of food.

We get our source of food from agricultural cultivation and mainly we grow or cultivate cereals like barley, *teff,* beans, and peas. My family had access to irrigation land but most of the time we cultivated potato only for consumption and selling.

Q2. What type of animal sources food do you have access and your consumption habit?

I did not eat eggs instead my family sold it to market for paying to different house hold expense. I eat eggs at time of begging and end of fasting ceremony.

Q3. What is healthy diet for you?

It is just a balanced diet which consists of vitamins, that prevents from illness. But, unhealthy diet like drinking alcohol.

Q4. Who get primary food from your family member?

In my family, first food was given for male child and fathers.

Q5. Does your family and peer influence your dietary choice?

“No” I am not influenced.

Q6. Do you get nutrition education at schools or village level?

No I do not get.

What is the barriers do not cultivate fruits and vegetables?

In my family, agro forestry plants do not cultivated, due to lack adaptation to local agro ecology, and lack of awareness in the community on it. The community plant equlptus tree rather than trees that

produce fruit like mango and avocado. In addition, we have limited irrigation land, agro forestry plant take long years to produce fruits as compared to potato which cultivated within 3 months.

In addition, the agricultural extension workers do not give support for farmer for cultivation of agroforestry plants that produce fruits.

In some session we only cultivate chills and carrot.

My daily meals during breakfast is brads, or injera with sauce made from mixed salt and red paper, lunch and dinner injera with wote.

In-depth interview -7 (Urban resident, female adolescents, age 16)

Q1. What is your food preference and eating habit?

I prefer to eat vegetables, *shero* (sauce made from legumes powder and eat with injera) and potato, from all I get easily -shero, sometimes I consumed milk, eggs, and meet.

Q2. What type of animal sources food do you have access and your consumption habit?

I get food items like milk, eggs, and meet occasionally.

At time of fasting I eat *shero*, the usual diet, at time of breakfast I eat *ferefe* which was prepared from potato and injera during fasting season. I eat eggs, meet and milk during none fasting times mainly during fasting season beginning and at the end. *Ferere* which is prepared from dry meet at time of none-fasting period, and potato ferefe at time of fasting

The family eat meat, milk and eggs during x-mass, epiphany, and new year.

My habitual diet at time of lunch and dinner *injera with wote* (sauce prepared from oil, salt, spices and legume powder) with injera (white teffe 75%, 25 % maize).

Q3. What is healthy diet for you?

For me healthy diet-eating is an eating habit of equal proportion carbohydrates, protein (body builder).

In-depth interview -8 (Urban resident, female adolescents, age 17)

Q1. What is your food preference and eating habit?

I prefer to eat vegetables, shero, I had breakfast eating habit during none fasting, but I do not eat at fasting time.

I eat diet at time fasting ferefer –which is prepared from injera made 50% teffe and 50% maize, and sauce made from onion and red paper. My habitual diet at lunch and dinner time was injera with wote.

Q2. What type of animal sources food do you have access and your consumption habit?

I eat eggs, especially during ceremonial times like new year, Chris-mass and epiphany when poultry meet was prepared. I drink milk up to 3 times per week during non-fasting periods. But, I have no appetite to eat meet in my home totally.

Q3. What is healthy diet for you?

In my views a food composition which consists of balanced food items which includes vegetables. Healthy diet means taking meals during each time.

Q4. Who get primary food from your family member?

There are no food preferences for male children at house hold level.

Q5. Does your family and peer influence your dietary choice?

I eat my meals together with other family member, there is no influence of family member on my food preferences. Totally, I do not face peers influence on my diet preferences.

Q6. Do you get nutrition education at schools or village level?

There is no planed nutrition education delivered at school levels.

Q7. Why do not you consume various food items?

Due to lack of market accessibility of variety of fruits and vegetable year round. In my town there is variation on accessibility of fruits and vegetables from time to time. There is limited vegetables during dry session and green vegetables are available at time of spring and up to mid of summer.

Q8. Do you have food consumption habit outside your home?

No, I have no eating habits outside my home.

In-depth interview -9 (Urban residence, female adolescents, age 15)

Q1. What is your food preference and eating habit?

I prefer food items like legumes, sometimes meet, but, I do not eat fat foods. In in addition I prefer to consume fruits like mango, orange and vegetable like cabbage, Lettuce and others.

Q2. What type of animal sources food do you have access and your consumption habit?

I have no access to animal source food like milk, eggs and meet, I and my family get from local market.

Q3. What type of food items you consumed at house hold level?

I and my family usual food habit is *shero wote with injera*, potato and sometimes we eat meet. I do not have eating habit of breakfast due to my mother do not prepare early in the morning and I do not get sufficient time to eat my breakfast. My mother had kids due this she did prepare breakfast in the morning. I eat eggs once per month or more infrequently by purchasing from local market.

Q3. What is healthy diet for you?

In my views healthy diet includes like vegetables, sometimes meet, legumes and fruits.

Unhealthy diet

Unhealthy diet such as excess sugar consumption, fats, oils and candy

I eat *injera with wote*, sometimes meet

Q4. Who get primary food from your family member?

In my family there is equal allocation of food items for all family members there is no food preferences for males and females.

Q5. Does your family and peer influence on your dietary choice?

I have no peer influence on my dietary habit

Q6. Do you get nutrition education at schools or village level?

I get nutrition education at school one per year in this year.

Q7. Why do not you consume various food items?

I do not get access to fed variety of foods in my family due to lack of diverse food items. they limit me to take almost similar food items.

In my locality there is market access of vegetables and fruits during spring and up to med of summer but banana is available throughout the year but it is not affordable in terms of cost.

Q8. Do you have food consumption habit outside your home?

No, I do not eat out of my home.

Q9. What is the cooking habit of food?

In-depth interview-10 (urban resident, female adolescent, age 17)

Q1. What is your food preference and eating habit?

I prefer mixed diet like cabbage and chills and other, I do not have desire to eat meet.

Some of the food items are fulfilled by family from market.

Q2. What type of animal sources food do you have access and your consumption habit?

I eat eggs –at beginning and end of fasting, where as I drink milk during none fasting time.

At time of lunch and dinner, I eat –shero (legumes, injera made from teffe).

Q3. What is healthy diet for you?

Eating vegetables

Unhealthy diet –consumption of fat food items.

Q4. Who get primarily food from your family member?

In my family there is no food preferences among family members.

Q5. Does your family and peer influence your dietary choice?

No, peer influence of my daily meal.

Q6. Do you get nutrition education at schools or village level?

No I do not get

Q7. Why do not you consume various food items?

I do not have good appetite for eggs. I do not eat snake eating habit, only I eat breakfast, lunch and dinner. In fasting time, I jumped breakfast.

Q8. Do you have food consumption habit outside your home?

No I do not eat

In-depth interview-11 (Urban resident, female adolescent, age 17)

Food preferences

I prefer to eat healthy diet like vegetables and shero, my family full file diet that I want.

Habitual diet –of family

I eat at breaking-fast shero with injera or lentils, carbohydrates *(mekorni and pasta)*.

I eat eggs, milk and meet at time of non-fasting time 1 or 2 times per weeks.

I eat at time fasting –mekorni, tea, injera with wote.

Healthy diet

Healthy diet including vegetables

Unhealthy diet –fat, high butter in wote

In my family I do not get special food items were not provided

I dislike milk and milk products, even eggs and meet

I do not eat outside home.

Habitual diet is shero with injera

We add small food at end of cooking

Some food items were given for father

No peer influence on my diet

I do not have food restriction habit for body image or sheep, but some students practice specially the urban students.

In-depth interview-12 (Urban resident, female adolescent, age -16)

Q1. What is your food preference and eating habit?

My habitual diet was injera with wote, sometime I eat cereals and vegetables.

I prefer –vegetable and fruits but my family do not full-fill.

My dietary habit is based on my family preference.

Q2. What type of animal sources food do you have access and your consumption habit?

In my family eggs and milk was given for young child, I eat with other family member during ceremony times. I dis-like to drink and eat milk and milk products because once up on a time I develop vomiting after drinking of milk. My habitual diet during breakfast was brad with tea, at lunch and dinner I eat injera with wote and sometimes we add vegetables in our dish.

Q3. What is healthy diet for you?

In my views healthy diet –consists of vegetables. Whereas unhealthy –meet. Our family restrict fat based meet.

Q4. Who get primarily food from your family member?

In my family primary food is given for young children.

Q5. Does your family and peer influence your dietary choice?

No peer influence on my feeding habit

Q6. Do you get nutrition education at schools or village level?

I get nutrition education on healthy diet from media in television; but we get limited education at school.

Q7. Why do not you consume various food items?

The reason that prevent me from consumption of vegetables was due to lack of sufficient income or money to purchase from market.

My family, and community does not give attention to a balanced diet, we eat what we found. Even I had animal source food items at my family level, our feeding habit is poor due to lack of awareness, and my family do not give attention to feed diversified food items for adolescents.

In my case I do not have food restriction habit for body image or sheep.

In urban community –our family provide different food items for our growth, But the rural community do not consider to feed child various mixed food items.

My family food sources were from market and we do not cultivate home gardening.

Mainly my feeding habit is *injera with wote*, and I may not get balanced nutrient for my growth.

Q8. Do you have food consumption habit outside your home?

I have no eating habit of outside home, but my peer eat food like packed biscuit and chips outside their home.

In-depth interview- farmer representative

In-depth interview-1 (Farmer, age 53 year)

Why not you practice agro forestry plantation to produce fruit and vegetables production in your locality?

Response –we do not involve in agroforestry fruit and vegetables production. In our area, we do not have suitable climatic condition for agroforestry fruit and vegetables production. besides we do not have knowledge on agroforestry plant production. In addition, animal destruction of these plants due to open grazing habit of the community.

One of the farmers said that; I believe I can get income from agroforestry plant cultivation but I had no land access because most of the agricultural land occupied by elder farmers.

I have animal like sheep and hens, but I do not have a habit of feeding of eggs and meet in my family. Because, I sold to the market to get money to pay for land rent to cultivated cereals food items and to buy agricultural impute like fertilizers.

Some farmers practice agroforestry plants cultivation for fruit production like mango, avocado but apple tree plantation was not practice and do not known by the local farmer. In contrary most of the farmer plants trees like equliptus in their farm land due to lack of knowledge on agroforestry plants.

There is lack of awareness and understanding of agroforestry plantation. Our agricultural habit is dominantly tradition ways. Even the agricultural extension workers do not fully support on how to cultivate locally adaptable fruit and vegetables. Now, this is a time of rainy season but almost all farmers cultivated equlptus tree. I have interest to cultivated agroforestry plants but I do not get access agro ecologically adaptable plants species (53, years old Farmer).

In-depth interview- 2 (farmer, age 32)

What is your producing habit of poultry?

I was working as chemistry teacher for 9 years, I start poultry production in recent time

Who are the primary customer of your poultry eggs and meet?

Mainly I sold my product to large town customer and for military camps. My primary customer was trader and few government workers. The local community buy egg from my poultry farms during ceremonial times and at the begging and end of fasting periods. The target family member who consume eggs are for under five children. My customers were not buy eggs for adolescents at all from my experience.

Almost all community cannot afford to buy eggs for adolescent and other family member feeding due to high cost of eggs. Now at this moment I sold on eggs for any customer seven (7) Ethiopian birr. The high cost of eggs and meet of poultry is due the increment of poultry production imputes like feeding and medicine.

Currently there was production of poultry eggs and meet in small scale and medium scale farming activity at all level but eggs and meet of poultry was no affordable for low income families. There is no existing local level poultry impute production like fodder, vitamin supplement and medicine that are required.

In-depth interview of Agricultural extension worker representative

In-depth interview -1 (Agricultural extension worker, 31 years)

Why farmer do not cultivate fruit and vegetables?

In my Keble there are three agro ecology Zones kola, degas and weyna degas agro ecology. The agro ecology Zone are dry and no access to irrigation. Some Keble's in kola agro ecology cultivate fruits and vegetables, but in degas agro ecology there is potential for apple production. But there is no access to plant apple tree in the area. No access of apple tree from woreda agricultural office. There is no non-governmental organization(NGO) support for woreda agricultural office. I request the worda agricultural office to bring apple tree but I do not get the tree to distribute for farmers. At summer time –farmers are engaged in planning eqlptus tree. This is due to equlptus tree is easily accessible at local area, in addition this tree does not need much water for growth.

We plane to plant discourse tree which has not value for fruit or vegetables it only used for soil and water conservation. The plant given for farmers feely without payment.

In-depth interview -2 (Agricultural extension worker, 29 years)

Why farmer do not cultivate fruit and vegetables?

In the area, farmers were engeged in traditional farming activities to cultivate cereals, and potatoes in rain feed agriculture methods. We try to promote farmers to cultivate fruit, and vegetables, but due lack of access to irrigation land and water. This traditional farming activity lead to availability of diversified food items in market.

In-depth interview education representatives

In-depth interview -1 (School director, age 38)

Q1-Is there any existing nutrition intervention which focus on adolescents?

Response- before one year there is one None governmental organization (NGO) which provide support vegetables seeds at school and community level for producing variety of food items. But due lack of water we do not cultivated at school levels and discontinued at this time.

In addition, this NGO started supplementation of weekly iron folic acid for adolescent girls at school level. But this weekly iron folic acid supplementation was not accepted by adolescents. This is due to lack of understanding the benefits of iron folic acid, wrong perception held on iron folic cause infertility, lack proper orientation and education to the students, lack of integration between school and health center or office. Besides the supplementation is targeted 10-19 years' female adolescents this leads to sense of discrimination.

Now at this movement there is no any school based intervention for adolescent's nutrition. Even though weekly iron folic acid supplementation was implemented in this school, but now the program was stopped due lack of coordination and support from health facility.

In-depth interview -2 (School director, age 31)

Q1-Is there any existing nutrition intervention which focus on adolescents?

There is no such strong school based nutrition intervention implemented in our school. Sometimes nutrition education was delivered by heath extension workers at school one or two times per year. The only services provided at school was deworming for intestinal parasite. There are no school gardening actives implanted at school level due to lack of water in school compound. In-depth interview -3 (School director, age 34)

Q1-Is there any existing nutrition intervention which focus on adolescents?

Totally, school health including nutrition intervention was not implemented due to lack of collaboration with health sectors. Some actives like school gardening actives were started in last year, but due to lack water the program do not continues at this movement. there are no nutrition education actives delivered by health professionals.

Adolescent meal observation at home level

Meal observer one (Female, diet observer data collectors, 27 years)

Meal type of adolescents

In morning traditional diet was eaten in three days of observation, the same eating habit was observed in lunch and dinner times. There are no vegetables, and fruit consumption as side dish darning meal times.

"The adolescent diet was almost in observed house hold consists of traditional diet injera with wote sauce in all meal times" (Female, diet observer data collectors, 27 years).

The same observation result was confirmed by second data collector "in my observation during all meal time of adolescents, there is no variation of diet" (male, diet observer, data collector, 31 year).

Observer two male, diet observer, data collector, 31 year

Type of food item eaten at time of breakfast from three days the adolescent does not eat in one day because of fasting. in second day she eats homemade bread and third day she eats injera with wote sauce. No habit of eating snake in all times. She was eating injera with wote at time of lunch and dinner during all days of observation.

Observer three (male, diet observer, data collector, 28 year)

In morning the adolescent goes to school with empty stomach due to fasting. then after school she eat traditional diet which are prepare cereals, which composed of injera with wote sauce, and cooked potato. At time of lunch and dinner similar diet was eaten. There was no fruit, vegetables and snack eating habit in all observation days.

In-depth interview Health sectors representatives

In-depth interview 1-woreda nutrition focal (age 48. Sex female)

What type of nutrition intervention delivered for adolescent in your woreda?

Ok, in my catchments population we provide nutrition services for under five children, and reproductive age women. In this woreda we focus on vitamin A supplementation and deworming for children. We also supplement iron folic acid for pregnant women. There are no nutrition intervention services specifically provided for adolescents.

Since I start my work as nutrition focal at Woreda Health office level "there is no planned program provided for adolescents at schools and community and house hold level, most of the time we provide nutrition services for under five childern and pregnant women (female woreda nutrition focal, age 48).

What are the major challenges you face to provide nutrition services for Adolescents?

In our context there lack of human power recruited in nutrition in health institutions. I am a BSc clinical nurse, the worda health office assign me as nutrition officer to collaborate in all health cents and posts. I work this as an addition duty with child health program. This over loaded duty on affected me to delivered nutrition intervention for adolescents, even for under five childern and reproductive age women. The other challenges were lack of clear guideline for adolescent nutrition intervention, lack of financial support, negligence of government for adolescent's nutrition in health system, and lack iron folic acid for segmentation.

In-depth interview 2-18-woreda Health Office Head (Male, age 34).

What type of nutrition intervention delivered for adolescent in your woreda?

There is no such planned and organized nutrition intervention for adolescents, some time we provide nutrition education during school health visit, and time of deworming activities. In addition, NGOs try to supplement weekly iron folic acid for adolescents in some schools in our worda, but currently this supplementation is stop due to lack of supply. Over in all health system nutrition intervention mainly focused on young children, under five childern, and reproductive age women.

Almost in all level of health facilities our nutrition intervention is focused on under five and reproductive age women, but adolescent nutrition overlooked (Male, Health office Head, age 34).

How was the implementation status of multi-sectorial nutrition for adolescents?

In our Woreda there is advocacy for multi-sectorial nutrition intervention approach in collaboration of health, education, agriculture, water supply, and others. This activity was written on paper, all sectors plane their annual activities, but there was no implementation at ground.

There is discussion and planning for implementation of multi-sectorial nutrition for adolescents, but there was no actual implementation ((Male, Health office Head, age 34).

What are the major challenges you face to provide nutrition services for Adolescents?

In my views there is low awareness about adolescent nutrition intervention in all health system leaders. The other barriers were lack of trained nutrition professional assigned in health facilities and leadership

level. This program was delivered with integration of child health at health center, and heath post level. Due to this most of the time the assigned professional had no knowledge, and skill on nutrition intervention to provide nutrition services for all segment of the population including adolescents. The other barriers were, there is no nutrition training manuals, and guide line which are specifically prepared for adolescents. In addition, there was low commitment from government side to allocate nutrition expert at all level even there is huge number of human nutrition graduate from universities. Most of the time nutrition activities were performed by other health professional like clinical nurse,

Midwife and other who are no sufficient knowledge to provide nutrition intervention.

In-depth interview -3- for regional Health office focal

What are the major challenges you face to provide nutrition services for Adolescents?

Here, the government assign 2 focal persons to coordinate nutrition intervention across the region. Imagine, how it is possible to follow and support each nutrition intervention in all segment of the population at regional level due to the over load. Up to know nutrition had no clear human structure and did no lead by nutrition professional at regional, woreda and health institution level. This is the key challenges that affect to implement for adolescents, children, and for pregnant mothers. In addition, there was week multi-sectorial collaboration for nutrition in between health, education, agricultural and other sectors.

We face challenges nutrition professional assignment to implement adolescents and other population at health system and community level due to clear structure at all level (regional nutrition focal, male, age 52).

In-depth interview -4- Federal level focal (Nutrition expert, Female, age 33)

What are the major challenges you face to provide nutrition services for Adolescents?

The Ethiopian minister of Health, provide nutrition intervention primarily for under two, five children, and reproductive age women mainly for pregnant mothers. The issue of adolescent's nutrition intervention overlook in health system. There were some initiatives at school level for promotion nutrition for adolescents. In past 30 years there is no clear and comprehensive adolescent's nutrition guideline for adolescents. But at this time there is development of guideline for adolescent's nutrition which consists of proven nutrition, assessment, nutrition specific and sensitive intervention in multi-sectorial approach.

The key challenges we face to implement nutrition intervention at all level of health system, there is clear structure developed by Ethiopian civil services to deploy nutrition professional in health institution, in all coordination structure, and other sectors like agricultures. The national nutrition program was coordinated under maternal and child health directors as nutrition unit. This poor structure leads to over look to implement proven nutrition specific, and sensitive nutrition intervention for adolescents, and other segment of population. Now a day the Ethiopian public and private universities, and college educate nutrition professional in undergraduate, and post graduate level up to PhD level. But there is no nutrition governing structure, even the existing unit level coordination at federal, and focal person at regional, Zonal and Woreda level are limited in number, most of them were not nutrition professional.

There is multi-sectorial nutrition intervention approach and from 2018 onwards Ethiopian food policy, Food and Nutrition strategy (FNS), multi-sectorial nutrition strategy, Ethiopian food based dietary guild line, and national nutrition program I and II were implemented. Other document like micronutrient deficiency prevention and control implementation guild line, adolescent nutrition guide line and infant young child and maternal nutrition guild line were under development. These and other nutrition and food related official document indicates their high level of government commitment to reduce malnutrition in all forms.

But food and nutrition strategy, and guides were not implemented at lower health system, and other sectors due lack of commitment, lack assigned nutrition human power, week multi-sectorial collaboration, and financial resources shortage (Nutrition expert, Female, age 33).
